# Erratum

**DOI:** 10.1111/eva.13380

**Published:** 2022-05-15

In the article by Bischofberger et al. (2021), the wrong image was inserted for Figure 1. The correct figure is as follows:

**FIGURE 1:**
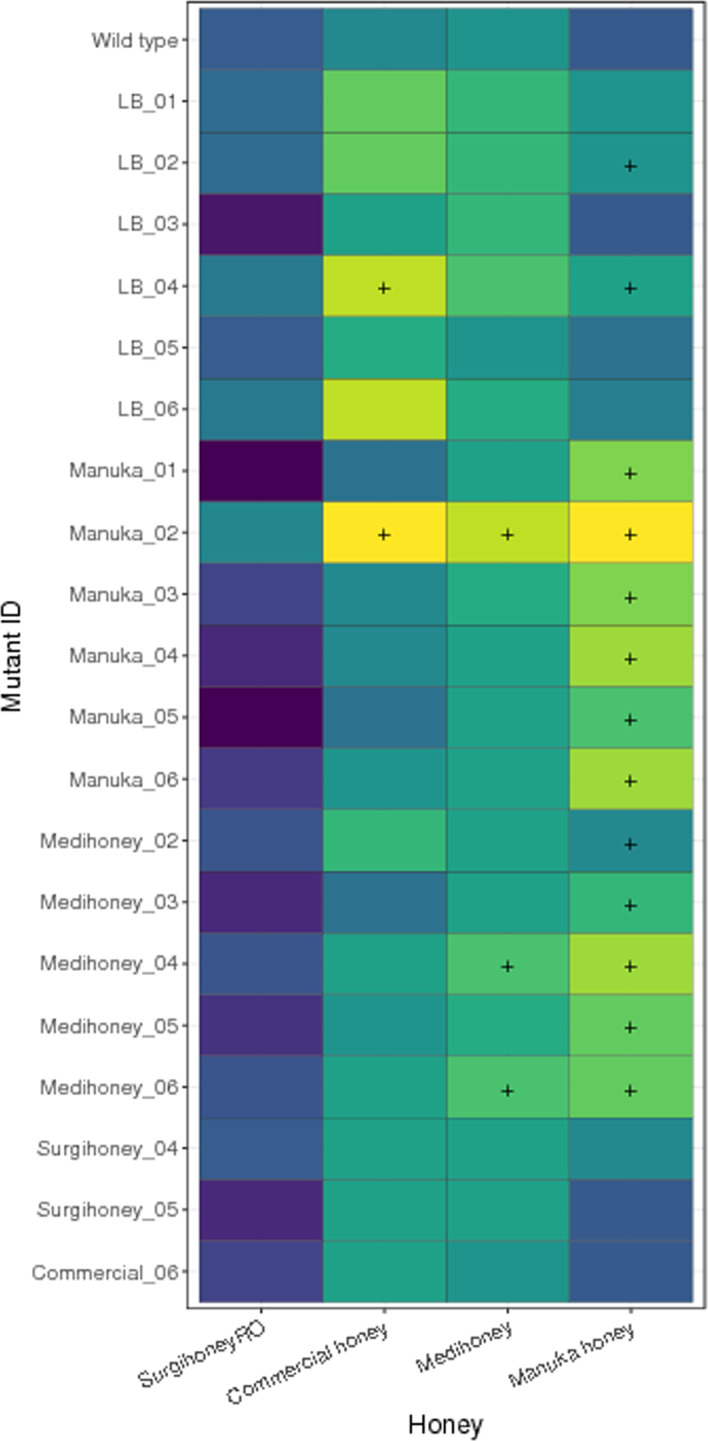
Susceptibility of serially passaged, putative resistant mutants with four different honey compounds. Each cell gives the median IC90 for a given isolate (the ancestral strain *Escherichia coli* K‐12 MG1655, in the top row, or a putative resistant mutant serially passaged with one of the four honey compounds, labelled according to compound and replicate selection line) assayed with one of the four honey compounds (columns). Each value is the median of four independent replicates; combinations where all replicates of a putative resistant mutant were higher than all replicates of the ancestor in the same treatment are indicated with a ‘+’. Individual replicates for each strain are shown in Figure S4
