# Supplementary figures and images for: Evolution of honey resistance in experimental populations of bacteria depends on the type of honey and has no major side effects for antibiotic susceptibility
Source: Evol Appl. 2021 Mar 10;14(5):1314–27. doi: 10.1111/eva.13200 (PMC8127710; doi:10.1111/eva.13200)

**A**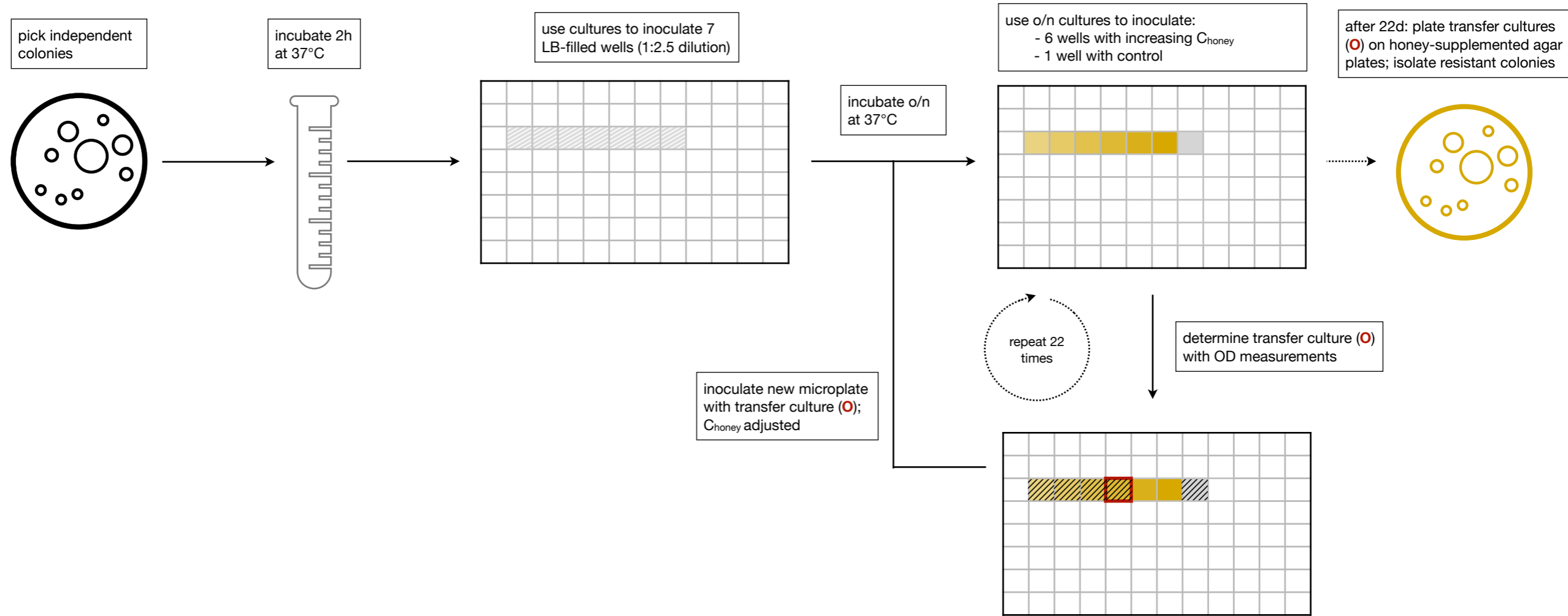**B**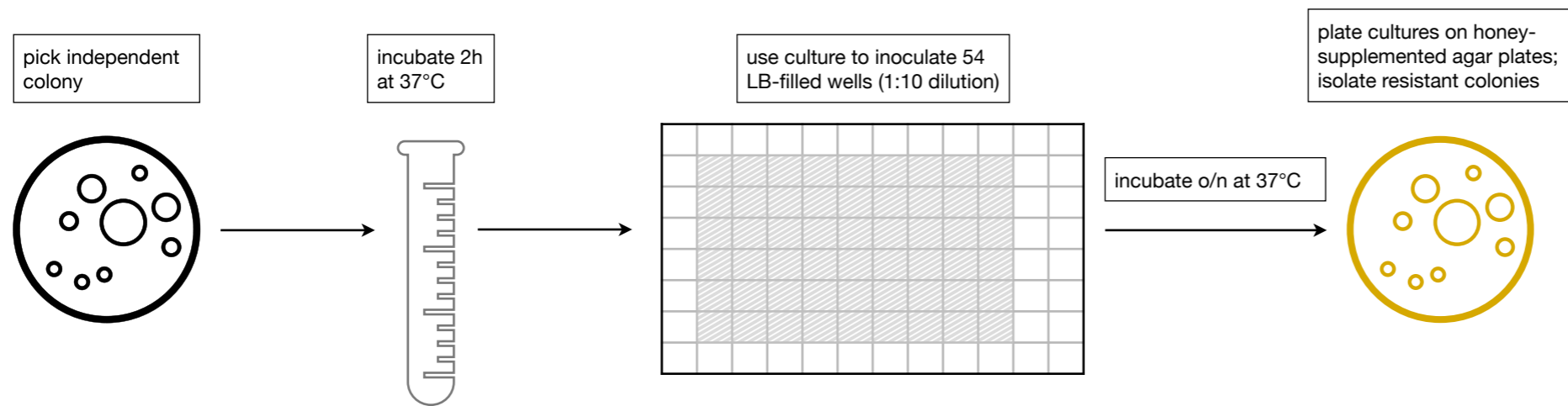

Supplement: Supplementary file 1 — Fig S1 [file EVA-14-1314-s003.pdf]
